# Biventricular thrombi of nonischemic cardiomyopathy

**DOI:** 10.11604/pamj.2014.17.210.4032

**Published:** 2014-03-16

**Authors:** Mohammed Muqeet Adnan, Syed Hashmi

**Affiliations:** 1 Department of Internal Medicine, University of Oklahoma Health Sciences Center, Oklahoma City, Oklahoma, USA

**Keywords:** Biventricular Thrombi, atrium, ischemia

## Image in medicine

47 year old male with history of alcoholic liver cirrhosis came in with severe bilateral lower extremity pain and new onset acute bilateral limb ischemia. Vitals at admission were stable and physical examination showed clear cut cool bilateral lower extremities below both ankle joints with all the 10 toes being black suggesting gangrene. Bilateral dorsalis pedis and Posterior tibial pulses were not felt and could not be detected with dopplers. A transthoracic Echocardiogram revealed that the patient had an Ejection fraction of 10-15% with diffuse hypokinesis, also were noted multiple biventricular thrombi with the largest in the left ventricle measuring 50×30mm in size extending from anterolateral papillary muscle upto the septal myocardium. Evaluation with a cardiac and aortic catheterization revealed non obstructive coronaries, complete occlusion of the bilateral anterior tibial, posterior tibial and peroneal arteries at the ankle level with zero flow below bilateral ankle joints. No intervention could be performed and hence the patient was at first anti coagulated with heparin and then bridged to Coumadin. Patient was discharged to follow up with orthopedic surgery for bilateral amputations. Biventricular thrombi are generally seen in patients with a pro thrombotic state like anti phospholipid antibody syndrome, heparin induced thrombocytopenia induced thrombosis, hypereosinophilic syndrome. Cases have been reported in patients with viral myocarditis and libman sacks endocarditis. It is generally very rare to see multiple biventricular thrombi in patients with low Ejection Fraction.

**Figure 1:**
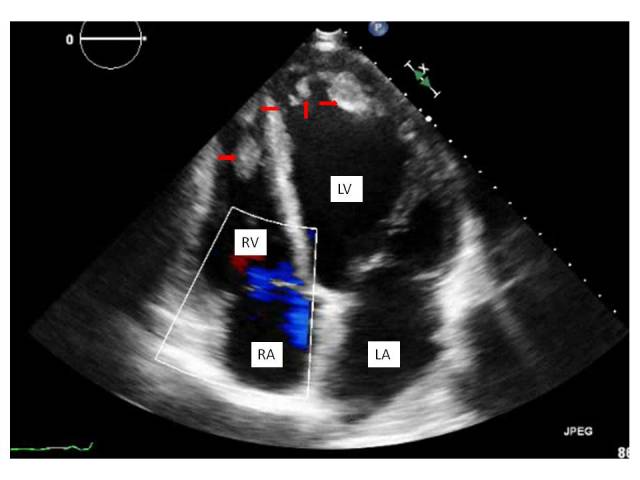
A 4-chamber view demonstrating a closer view of a large Thrombus

